# Human Macrophages Clear the Biovar Microtus Strain of *Yersinia pestis* More Efficiently Than Murine Macrophages

**DOI:** 10.3389/fcimb.2019.00111

**Published:** 2019-04-24

**Authors:** Qingwen Zhang, Youquan Xin, Haihong Zhao, Rongjiao Liu, Xiaoqing Xu, Yanfeng Yan, Zhipeng Kong, Tong Wang, Zhizhen Qi, Qi Zhang, Yang You, Yajun Song, Yujun Cui, Ruifu Yang, Xuefei Zhang, Zongmin Du

**Affiliations:** ^1^Qinghai Institute for Endemic Disease Prevention and Control, Xining, China; ^2^State Key Laboratory of Pathogen and Biosecurity, Beijing Institute of Microbiology and Epidemiology, Beijing, China

**Keywords:** *Yersinia pestis*, host-specific pathogenicity, biovar microtus, human lymphocyte, transcriptomes

## Abstract

*Yersinia pestis* is the etiological agent of the notorious plague that has claimed millions of deaths in history. Of the four known *Y. pestis* biovars (Antiqua, Medievalis, Orientalis, and Microtus), Microtus strains are unique for being highly virulent in mice but avirulent in humans. Here, human peripheral lymphocytes were infected with the fully virulent 141 strain or the Microtus strain 201, and their transcriptomes were determined and compared. The most notable finding was that robust responses in the pathways for cytokine-cytokine receptor interaction, chemokine signaling pathway, Toll-like receptor signaling and Jak-STAT signaling were induced at 2 h post infection (hpi) in the 201- but not the 141-infected lymphocytes, suggesting that human lymphocytes might be able to constrain infections caused by strain 201 but not 141. Consistent with the transcriptome results, much higher IFN-γ and IL-1β were present in the supernatants from the 201-infected lymphocytes, while inflammatory inhibitory IL-10 levels were higher in the 141-infected lymphocytes. The expressions of CSTD and SLC11A1, both of which are functional components of the lysosome, increased in the 201-infected human macrophage-like U937 cells. Further assessment of the survival rate of the 201 bacilli in the U937 cells and murine macrophage RAW 264.7 cells revealed no viable bacteria in the U937 cells at 32 hpi.; however, about 5–10% of the bacteria were still alive in the RAW264.7 cells. Our results indicate that human macrophages can clear the intracellular *Y. pestis* 201 bacilli more efficiently than murine macrophages, probably by interfering with critical host immune responses, and this could partially account for the host-specific pathogenicity of *Y. pestis* Microtus strains.

## Introductions

*Yersina pestis*, the causative agent of plague, is responsible for three historical pandemics including the notorious Black Death in medieval Europe (Perry and Fetherston, 1997; Butler, 2009). This lethal pathogen manifests itself as three main clinical forms: bubonic plague, pneumonic plague and septicemia plague (Perry and Fetherston, 1997). Pneumonic plague, the most serious form of the disease, leads to the certain death of victims if not properly treated in time. *Y. pestis* is a highly contagious pathogen that can spread quickly among closely contacted individuals by airborne droplets, thereby posing the potential threat of disease outbreaks in populations under suitable circumstances, which could include bioterrorism (Inglesby et al., 2000). Different *Y. pestis* strains are classified into four biovars (Antiqua, Mediaevalis, Orientalis and Microtus) according to their phenotypic properties (Zhou et al., 2004). Since the announcement of whole genome sequences of *Y. pestis* CO92 (biovar Orientalis) and KIM (biovars Mediaevalis) (Parkhill et al., 2001; Deng et al., 2002), the whole genome sequence data for more than 30 different *Y. pestis* strains, including each of the four biovars, have been released so far (https://www.ncbi.nlm.nih.gov/). Biovar Microtus was recently proposed to be a novel *Y. pestis* biovar because strains isolated from Microtus-related plague foci in China are lethal to microtus and other small rodents but avirulent to larger mammals and human (Fan et al., 1995), and unlike strains belonging to the three classical biovars, those strains can reduce nitrate, ferment rhamnose and melibiose, but not arabinose (Zhou et al., 2004). Biovar Microtus strains are distributed in the *Microtus brandti* plague focus of the Xilin Gol Grassland and the *M. fuscus* plague focus of the Qinghai-Tibet Plateau in China. However, since the first isolation of *Y. pestis* strains in the 1970s from the Microtus-related foci in China, no cases of human plague have been reported to be linked to these strains although serious enzootic plague epidemics have occurred every few years in the same areas. Human volunteers were subcutaneously inoculated with 1.5 × 10^7^ colony forming units (CFU) of the *Y. pestis* strains isolated from Microtus-related foci and the results confirmed for the first time that these strains are avirulent in humans (Fan et al., 1995).

It has been established that *Y. pestis* has evolved recently from *Y. pseudotuberculosis* and only limited genetic diversity has been found among the different *Y. pestis* strains (Achtman et al., 1999, 2004). The genome composition of the different *Y. pestis* biovars are very similar, although there is evidence of frequent events of gene rearrangement, large gene fragments deletion, gene loss, and gene inactivation among those genomes (Song et al., 2004). However, despite the monumental advances in comparative genomics and proteomics that have taken place over recent years, we are no further in elucidating the mechanisms underlying the host-specific pathogenicity of the Microtus strains (Song et al., 2004; Zhou et al., 2012). Therefore, in the present study, human peripheral lymphocytes were infected with the fully virulent biovar Antiqua strain 141 or the human avirulent Microtus strain 201, and their transcriptomes were compared. We observed significantly different responses in, for example, the cytokine-cytokine receptor interaction pathway, chemokine signaling pathway, Toll-like receptor (TLR) signaling pathway, and lysosome pathways between the 201- and 141- infected lymphocytes, suggesting that the ability of human lymphocytes to restrict infection caused by strain 201 or 141 might be very different. IFN-γ and IL-1β, which are recognized to benefit the host defenses against plague, were present at significantly higher levels in the supernatant from the 201-infected lymphocytes, while levels of the inhibitory IL-10 inflammatory cytokines were higher in 141-infected lymphocytes. Further assessment of the survival of 201 bacilli in human macrophage-like U937 cells and murine macrophage RAW 264.7 cells revealed that human macrophages clear intracellular *Y. pestis* 201 bacilli more efficiently than mice macrophages do. Our results showed that human macrophages exhibit much higher bactericidal activities than mouse macrophages and this finding might partially account for the host-specific pathogenicity of *Y. pestis* Microtus strains.

## Results

### Overview of the Comparative Transcriptome of Human Peripheral Lymphocytes Infected With Different *Y. pestis* Biovars Strains

Human peripheral lymphocytes were infected with the human-avirulent Microtus 201 strain or the fully virulent Antiqua strain 141 each at a multiplicity of infection (MOI) of 10. The infected lymphocytes harvested at 2, 4, and 8 h post-infection (hpi) were subjected to the total RNA isolation and RNA sequencing (RNA-seq) analysis. Clean reads from the RNA-seq analysis were mapped to the annotated human genome (NCBI36/hg18). Each library comprised around 4.5 × 10^7^ clean reads, 62.83% of which were on average mapped uniquely to the human reference genome (Table 1). Gene expression was calculated using the fragments per kilobase of gene per million fragments mapped (FPKM) method (Mortazavi et al., 2008), and the genes whose expression levels were altered by over 2-fold in expression level with false discovery rates (FDRs) ≤ 0.001 were defined as being the differentially expressed genes (Benjamini and Yekutieli, 2005).

**Table 1:** RNA-seq library descriptions.

**ID**	**Sample name**	**Clean reads^a^**	**Unique match (percent)^b^**	**Total unmapped reads (percent)^c^**	**Expressed gene**
1	141-2h	44641336	29202733 (65.42%)	13240983 (29.69%)	15462
2	141-4h	45138004	29179029 (64.64%)	13934344 (30.87%)	15477
3	141-8h	44789942	28803453 (64.31%)	14196597 (31.70%)	15568
4	201-2h	44201980	28907751 (65.40%)	13441594 (30.41%)	15547
5	201-4h	44294928	27656449 (62.44%)	13662809 (30.85%)	15506
6	201-8h	44851572	27616280 (61.57%)	14148314 (31.54%)	15570
7	Normal	44701980	28449706 (63.64%)	13266250 (29.68%)	15512

a Clean reads represent the number of high quality clean reads.

b Unique matches denotes the reads that mapped to unique positions in the reference human genome.

c Total unmapped reads denotes the number of reads that couldn't be mapped to the reference genome.

*The percentages of the unique matched reads and the unmapped reads were calculated by dividing the values of the unique or unmapped reads by those of the clean reads of clean reads × 100 (values shown in brackets)*.

**Figure 1:**
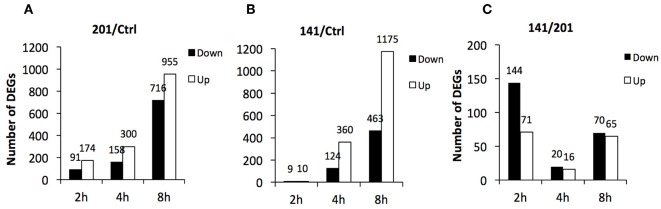
Differentially expressed genes in human peripheral lymphocytes infected with *Y. pestis* 201 or 141 strains. Numbers of genes that were differentially expressed at 2, 4, and 8 hpi in 201-infected **(A)** or 141-infected lymphocytes **(B)** in comparison with the uninfected lymphocytes and between the 201-infected and 141-infected lymphocytes **(C)** are shown here.

First, the transcriptomes of the peripheral lymphocytes infected with strains 201 or 141 were compared with that of the uninfected lymphocytes, respectively. Differentially expressed genes were identified according to the aforementioned criteria and the numbers of genes at each time point post-infection were calculated. More than 260 genes were differentially expressed at 2 hpi between the 201-infected and uninfected lymphocytes, and the number increased greatly as the infection period proceeded and exceeded 1,600 at 8 hpi (Figure 1A). In sharp contrast, the transcriptome of the 141-infected peripheral lymphocytes differed very little from that of the uninfected cells at 2 hpi, and fewer than 20 genes were found to be differentially expressed, although the numbers of differentially expressed genes at 8 hpi in the 141- and 201-infected cells were high and comparable (Figure 1B).

Further comparative analysis showed once again that the most remarkable gene expression changes between the 201- and 141-infected peripheral lymphocytes occurred at the earliest time point that was analyzed (Figure 1C). The differentially expressed genes were relatively fewer at 8 hpi although they were quite abundant in the 201-infected (Figure 1A) and the 141-infected (Figure 1B) lymphocytes in comparison with the uninfected lymphocytes control. These data demonstrate that the initial contact by the human peripheral lymphocytes with the two *Y. pestis* strains triggered two obviously distinct responses, one of which could possibly facilitate efficient recognition of 201 bacilli and boost strong immune responses, while the other failed to do so in the case of infection with *Y. pestis* strain 141. It appeared that the 141-infected lymphocytes were strongly inhibited in their ability to deploy an inflammatory immune response because of a vital virulence mechanism present in strain 141, but absent in strain 201, which might originate from some as yet uncharacterized difference(s) between the two strains.

To validate the RNA-seq data, primer pairs for 23 genes were designed for quantitative RT-PCR analysis (Supplementary Table 1), among which 12 genes were up-regulated, 7 genes down-regulated and 3 unchanged in the 201-infected lymphocytes in comparison with the uninfected control according to the RNA-seq results. qRT-PCR was performed using the same RNA samples used in RNA-seq library construction as templates, 16s RNA was the reference gene for data normalization, and the log2 ratios of the target gene concentrations in the 201-infected lymphocytes to those of the uninfected control were calculated. The correlation efficiencies (R^2^) between the qRT-PCR and RNA-seq analysis results were over 0.90 for the RNA samples collected at 2 and 8 hpi, and 0.83 for the samples collected at 4 hpi (Supplementary Figure 1 and Table 2). These results indicate that the RNA-seq data described in this study are highly reliable.

**Table 2:** Pathway enrichment analysis of the differentially expressed genes in 201- or 141-infeced peripheral lymphocytes.

**Pathway term**	**201-infected**			**141-infected**		
	**2h**	**4h**	**8h**	**2h**	**4h**	**8h**
hsa04060:Cytokine-cytokine receptor interaction	37	29	70		34	76
hsa04620:Toll-like receptor signaling pathway	13					
hsa04062:Chemokine signaling pathway	16		40		16	45
hsa04630:Jak-STAT signaling pathway	12	16			19	
hsa04640:Hematopoietic cell lineage	9		28			28
hsa04142:Lysosome			30			
hsa04666:Fc gamma R-mediated phagocytosis					11	
hsa04512:ECM-receptor interaction			22		10	
hsa04621:NOD-like receptor signaling pathway			18			21
hsa04672:Intestinal immune network for IgA production			15			16
hsa05330:Allograft rejection			12			
hsa05310:Asthma						13

*Pathway enrichment analysis was performed using DAVID 6.7 bioinformatics tool (http://david.abcc.ncifcrf.gov) (Huang da et al., 2009). Only pathways that were significantly (p < 0.05, Fisher's exact test followed by the Bonferroni multiple testing correction) enriched at any time points in the 201- or 141- infected lymphocytes are shown here. Numbers in the table represents the differentially expressed gene numbers in a specific pathway at the indicated time point*.

### Multiple Immune Response Pathways Differ in Their Responses to Infection With Strains 201 or 141

**Figure 2:**
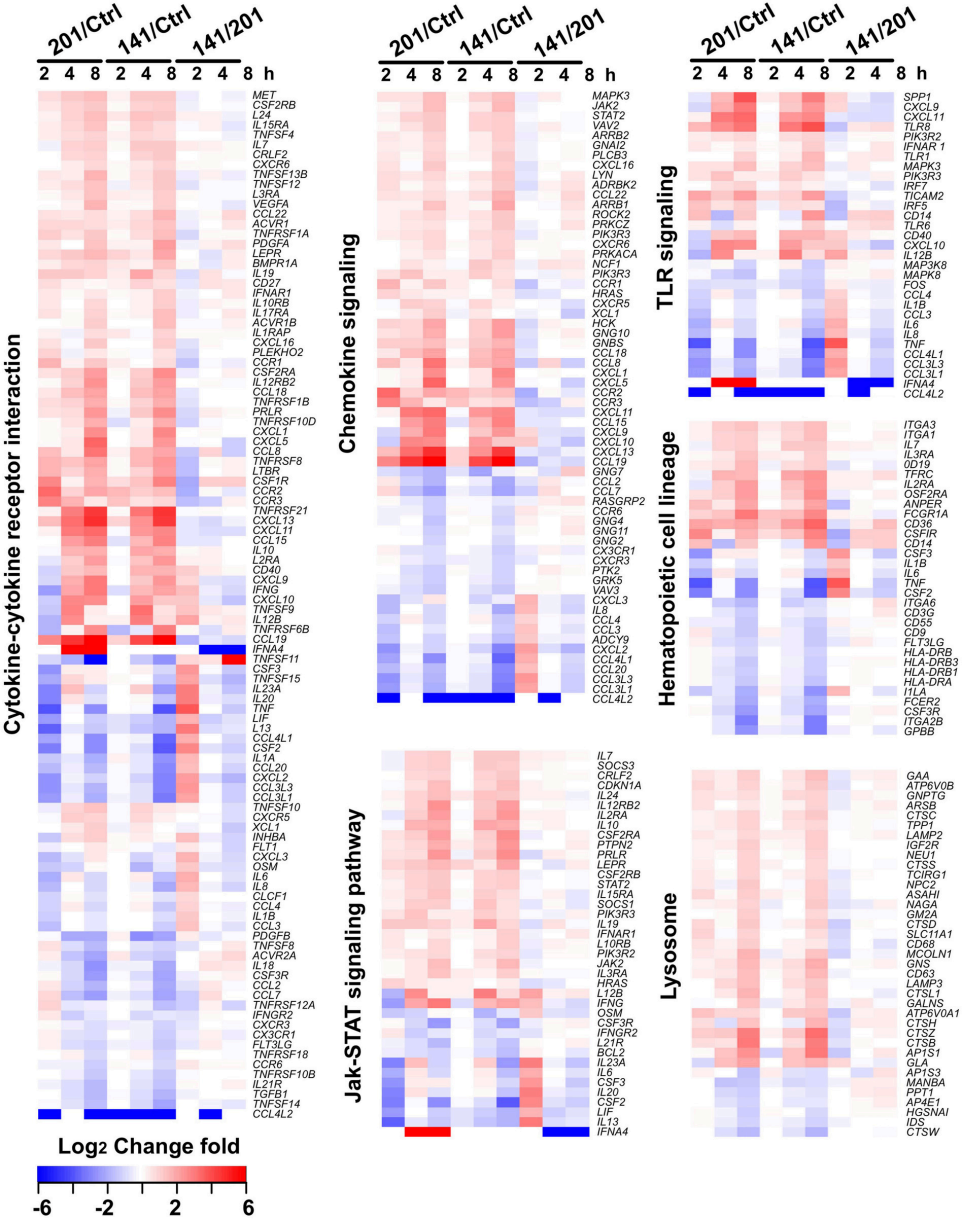
Gene expression patterns of the pathways that showing the significant differences between the *Y. pestis* 201- and 141-infected human peripheral lymphocytes. Some of the critical cellular pathways that were significantly enriched in the genes differentially expressed at 2, 4, or 8 hpi in 201- and 141-infected lymphocytes are shown (*p* < 0.05, Fisher's exact test followed by the Benjamini multiple testing correction). Changes of the differentially expressed genes are presented in different colors as shown in the color key.

To characterize the host transcriptomic response to the human avirulent Microtus strain 201, genes differentially expressed in 201- or 141-infected human peripheral lymphocytes, compared with the uninfected cells, were subjected to the pathway enrichment analysis using the DAVID 6.7 bioinformatics tool (http://david.abcc.ncifcrf.gov) (Huang da et al., 2009). Consistent with the observation that the genes differentially expressed between 201- and 141-infected lymphocytes peaked at 2 hpi, several pathways critical for inducing the intense immune responses required for invading pathogen clearance were significantly enriched in the 201- but not the 141-infected lymphocytes (*p* < 0.05, Fisher's exact test followed by the Bonferroni multiple testing correction) at this time point. These pathways included the cytokine-cytokine receptor interaction pathway, the chemokine signaling pathway, the TLR signaling pathway, and the Jak-STAT signaling pathway, among others (Table 2). Figure 2 shows the heat maps for some of the differentially enriched pathway in details. Strikingly, at the later infection time points (4 and 8 hpi), the pathway enrichment analysis revealed a significant difference between the 201- and 141-infected lymphocytes in the lysosome pathway, the Fcγ receptor-mediated phagocytosis pathway and the extracellular-matrix (ECM) receptor pathway, suggesting that the phagocytosis and lysosome maturation process could be differentially modulated in the lymphocytes infected with the two different strains.

Pathogens invading the mammalian host are recognized by pathogen recognition receptors (PRRs) via sensing of the conserved ligands on microorganisms (Takeuchi and Akira, 2010), and TLRs represent the major PRRs responsible for bacterial pathogen recognition (Krishnan et al., 2007). The signaling pathways triggered by TLRs exhibit distinct characteristics in several key molecules (Supplementary Figure 2A). CD14 protein binds to lipopolysaccharide (LPS), which after transfer to the TLR4/MD-2 complex triggers the downstream signaling required for the production of pro-inflammatory cytokines and type I interferon (Leeuwenberg et al., 1994; Ishihara et al., 2004). TLR adaptor molecule 2 (TICAM2) is an adaptor that transduces signals upon ligand binding to TLR4. Interferon regulatory factor 5 (IRF5) is a transcriptional factor involved in activation of interferon and the immune system. TLR8 recognizes single-stranded RNA from both viruses and bacteria (Sarvestani et al., 2012). The expression levels of *CD14, TLR8, TICAM2*, and *IRF5* were much higher in the 201-infected lymphocytes than in their 141-infected counterparts at 2 hpi, a finding further confirmed by the qRT-PCR analysis (Supplementary Figure 2B), although their expression levels after this time point gradually became comparable in the 201- and 141-infected lymphocytes.

As a facultative intracellular pathogen, *Y. pestis* is readily taken up by phagocytes but the pathogen becomes phagocytosis-resistant shortly after its initial intracellular survival and replication (Pujol et al., 2009; Connor et al., 2015). Thus, in systemic infection of the mammalian hosts, the survival of *Y. pestis* in host macrophages at the early stage of infection are critical for the later disease progress. Our results revealed that the differentially expressed genes were significantly enriched in the lysosome pathway in 201-infected lymphocytes, and 30 genes were significantly changed at 8 hpi (Table 2) but not in the 141-infected cells. Although we saw no significant enrichment in this pathway at 2 hpi, several key genes (e.g., *ATP6V0A1, CYSZ, CTSD* and *SLC11A1*) involved in lysosome formation and maturation were expressed at higher levels in the 201-infected lymphocytes at 2 hpi than in the 141-infected cells. *ATP6V0A1* encodes a vacuolar ATPase that mediates acidification of intracellular organelles, and vacuolar acidification is necessary for zymogen activation in the lysosome (Saw et al., 2011). CTSD protein exhibits pepsin-like activity, whereas CTSZ is a lysosome cysteine protease that has been shown to have lysosomal degradative capacity (del Cerro-Vadillo et al., 2006; Liu et al., 2014; Tranchemontagne et al., 2016). SLC11A1 functions as a divalent transition metal transporter involved in host resistance to infection with *Mycobacterium tuberculosis* and *M. leprae*, and also is associated with some inflammatory diseases (Govoni et al., 1999). qRT-PCR analysis of the aforementioned genes expressed in the 201- and 141-infected lymphocytes (Supplementary Figure 3) confirmed the RNA-seq results that the genes were expressed at higher levels in the 201-infected lymphocytes than in their 141-infected counterparts, suggesting that the lysosome pathway response differed between the infection with strains 201 and 141.

### Cytokine Secretion in Human Lymphocytes Infected With *Y. pestis* Strain 201 Differ Significantly From Those Infected With Strain 141

**Figure 3:**
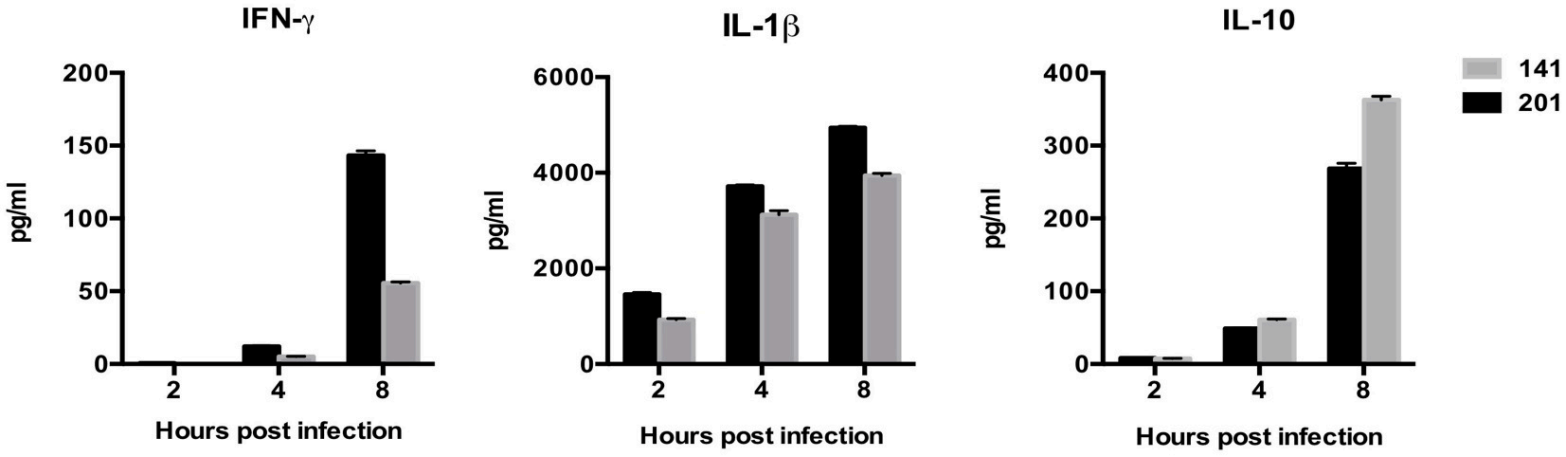
Human peripheral lymphocytes infected with the *Y. pestis* 201 strain secreted more abundant IFN-γ and IL-1β, and less immunosuppressive IL-10 than those infected with the 141 strain. Culture supernatants from the infected lymphocytes were collected at 2, 4, and 8 hpi and analyzed for the different cytokines using BD^TM^ CBA Kits. Significantly higher IFN-γ and IL-1β levels were present in the supernatant from the 201-infected lymphocytes, while inflammatory inhibitory IL-10 was higher in the 141-infected lymphocytes at 8 hpi. Figures were drawn using Graphpad Prism 5.0, and the statistical significance of the differences in the cytokine levels between the different groups of samples were analyzed by two-way ANOVA analysis followed by Bonferroni post-tests.

Next, we sought to compare cytokines and chemokines secretion in lymphocytes infected with the two different strains. Culture supernatants from the infected lymphocytes were collected and analyzed for IL-8, IL-1β, IL-6, IL-10, TNF-α, IL-12p, Interferon-γ (IFN-γ), IL-17A using BD^TM^ CBA Kits. Only the cytokines and chemokines that showed statistically significant differences (*p* < 0.05) were taken into account in the following analysis (Figure 3). The immunological measurements results showed that the 201-infected lymphocytes secreted much more IFN-γ and IL-1β than those infected with strain 141. IFN-γ is critical for protection against plague and treatment with IFN-γ greatly enhances the bactericidal activity of macrophages against the *Y. pestis* bacterium (Pujol et al., 2005; Parent et al., 2006). *Y. pestis* is known to be able to suppress the production of inflammatory cytokines IL-1β to promote its infection process (Ratner et al., 2016). Production of IFN-γ and IL-1β, both of which paly essential roles in host immune responses, appear to be significantly inhibited in the presence of strain 141 in comparison with the human avirulent strain 201. By contrast, secretion of the immunosuppressive cytokine Interleukin-10 (IL-10) was significantly higher in the 141-infected lymphocytes, unlike their 201-infected counterparts, which accords with previous reports that IL-10-deficient mice are resistant to *Y. pestis* (Turner et al., 2009).

### Expression Analysis for Three Essential Proteins in Functional Lysosome in U937 and RAW264.7 Cells Infected With the 201 Strain

**Figure 4:**
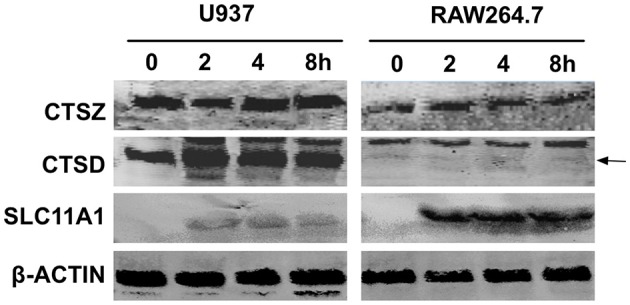
Immunoblotting detection of several proteins involved in lysosome activation in U937 and RAW264.7 cells infected with the *Y. pestis* 201 strain. Human macrophage-like U937 cells primed with PMA and murine macrophage RAW264.7 cells were infected with *Y. pestis* strain 201, and the cells were collected at 2, 4, 8 hpi and lysed for the immunoblotting detection of SLC11A1, CTSD, and CTSZ using specific antibodies. At least three independent experiments were performed and a representative result was shown here. The arrow indicates the position of CTSD bands.

The immediate up-regulation of the proteins involved in lysosome activation in 201-infected human lymphocytes promoted us to determine their expression levels after infection with *Y. pestis* strain 201. Human macrophage-like U937 cells primed with phorbol myristate acetate (PMA) and murine macrophage RAW264.7 cells were infected with *Y. pestis* strain 201, and the infected cells collected at 2, 4, 8 hpi were lysed for the immunoblotting detection of ATP6V0A1, SLC11A1, CTSD and CTSZ. Primed U937 cells possess major macrophage characteristics and can therefore resemble lysosome activation quite well in the infected host phagocytes (Koren et al., 1979; Grabenstein et al., 2006). With the exception of ATP6V0A1, whose lack of detection was probably related to its low abundance, all of the other three proteins were successfully detected (Figure 4). The levels of CTSD seemed to be slightly increased in the U937 cells after infection with the 201 strain, and the levels of CTSZ showed no significant alterations during the infection. Because experiments involving human virulent *Y. pestis* strains must be performed at biosafety level 3 laboratory, we were unable to obtain the expression levels of these proteins in 141-infected lymphocytes. However, interestingly, when the RAW264.7 cells were infected with *Y. pestis* strain 201, both the basal expression of CTSD and those after infection were quite low compared with that of the U937 cells, indicating that very low expression of this protease occurred under these conditions. The expression levels of SLC11A1 in both U937 and RAW264.7 cells were significantly increased after infection, and the induction of SLC11A1 in RAW264.7 seemed to be even stronger. The different expression patterns of CTSD and SLC11A1 in U937 and RAW264.7 hinted us that the lysosome activity toward *Y. pestis* strain 201 could be distinctive, which is likely relevant to the host-specific pathogenicity of Microtus strains of *Y. pestis*. However, no significant alteration in CTSZ expression was seen in both U937 and RAW264.7 cells although its' transcript level showed > 10-fold abundance at 8 hpi in the human lymphocytes, probably related to some post-transcriptional regulatory mechanisms.

### The Survival Rate of *Y. pestis* Strain 201 Is Significantly Lower in U937 Cells Than in RAW264.7 Cells

**Figure 5:**
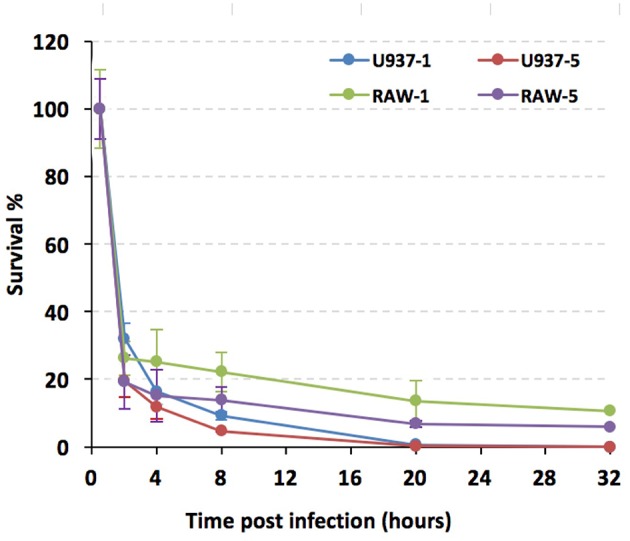
*Y. pestis* strain 201 survival rates were significantly lower in U937 than in RAW264.7 cells. PMA primed U937 and RAW264.7 cells were infected with *Y. pestis* 201 strain at an MOI of 1 or 5, and the living bacteria inside the cells at 2, 4, 8, 20, and 32 hpi were counted by plating the cell lysates onto the agar plates. Experiments were performed in triplicates for three independent times and the similar results were obtained. The survival percentages of the bacteria, as based on a representative result, are shown in average and standard deviation.

Because U937 and RAW264.7 cells seemed to respond to 201 and 141 strains differentially in respect of lysosome activation, we wanted to further characterize the interactions of *Y. pestis* strain 201 with these cell lines. U937 and RAW264.7 cells were infected with the bacteria and their survival percentages were measured using a gentamycin protection assay followed by plating cell lysates on agar plates. The U937 cells were stimulated with PMA (100 ng/mL) for 48 h before they were infected. Both U937 and RAW264.7 cells were infected with the *Y. pestis* 201 strain at a MOI of 1 or 5, and the infections were allowed to proceed for 32 h. The number of live bacteria in U937 and RAW264.7 cells at 0.5 h hpi were designated as the initial value and the survival percent at 2, 4, 8, 20, and 32 hpi were calculated by dividing the corresponding live bacterial cell number to that at 0.5 hpi. The percentage survival of *Y. pestis* bacilli decreased drastically to about 25–30% in both types of cells at 2 hpi at a MOI of 1. This percentage was sustained in RAW264.7 cells at a relatively steady level, dropping only 4% from 2 to 8 hpi; however, the bacteria inside the U937 cells were cleared much faster, as shown by the prominent decrease from 32 to 9% during this time period. At 32 hpi, no living 201 bacteria were detected in the U937 cells, whereas 10.6% of the bacteria were still alive in the RAW264.7 cells (Figure 5). When the cells were infected with *Y. pestis* 201 at an MOI of 5, similar results were found, where the percentage survival of the 201 bacteria in the RAW264.7 cells was significantly higher than that in the U937 cells, indicating that human macrophages can clear the *Y. pestis* Microtus strain more efficiently than the murine macrophages.

## Materials and Methods

### Cell Cultures, Bacterial Strains and Infections

Peripheral lymphocytes were isolated from the whole blood collected from 8 healthy donors using Ficoll-Paque Plus (GE Healthcare, USA). The purified lymphocytes were maintained in RPMI-1640 medium supplemented with 10% fetal bovine serum (FBS) and 2 mmol/L of L-glutamine at 37°C in a 5% CO_2_ incubator. *Y. pestis* 141 strain is a biovar Antiqua strain, highly virulent in mice and human. *Y. pestis* strain 201 belongs to the biovar Microtus and is highly virulent in mice but avirulent in humans. The glycerol-preserved strains were inoculated into 5 mL of brain heart infusion (BHI) medium and allowed to grow at 26°C for 24 h. The strains recovered were subjected to three consecutive passages and the third passage cultures were grown until they approached an optical density the (OD) _600nm_ value equal to 1. Bacterial cultures were centrifuged and resuspened in RPMI-1640. The purified human peripheral lymphocytes were infected with the bacterial suspensions of 141 or 201 strains at an MOI of 10, and the infected cells were centrifuged briefly to promote the adhesion of the bacteria to the lymphocytes before incubation at 37°C in a 5% CO_2_ incubator. Gentamycin at 100 μg/ml was added after 0.5 h of infection to kill the extracellular bacteria in order to inhibit the proliferation of the bacteria during infection. At the 0.5, 2, 4, and 8 hpi, the culture supernatants from the 201- or 141-infected lymphocytes were collected and centrifuged at 4.000 rpm for 5 min to remove the floating bacteria and cell debris, and the cleared supernatants were stored in small aliquots for the cytokine measurements. The infected lymphocytes were collected and subjected to total RNA isolation.

### RNA Isolation, Sequencing and Data Analysis

Total RNAs were purified from the lymphocytes infected with strains 201 or 141 using a TRIzol reagent (Invitrogen, Carlsbad, CA) and the isolated RNAs were treated with RNAse free DNase I (Thermo Fisher Scientific, Waltham, MA). The RNA integrity number, rRNA ratio (28S/18S), and the total RNA concentration in the samples were determined according to the previous descriptions (Du et al., 2014; Yang et al., 2017). Sequencing libraries were prepared using the Illumina Truseq RNA sample preparation kit according to the manufacturer's protocol. Briefly, the first-strand cDNA was generated by First Strand Master Mix and SuperScript II reverse transcription using random primers and the second-strand cDNA was synthesized using DNA polymerase I and RNase H. The fragmented cDNA molecules were end-repaired, purified, and then ligated with PloyA. The sequencing was performed using Illumina HiSeq 2000 (Illumina Inc., USA). The original image data is converted into “raw reads” via base calling. To obtain clean reads of acceptable quality, raw data filtering and quality control were performed to remove the adaptors-containing reads, unknown bases amounting to more than 10%, or low quality bases. The Burrows-Wheeler alignment tool was used to map the clean reads to the human reference genome (NCBI36/hg18) (Li and Durbin, 2009), and Bowtie was used to map to the reference genes (Langmead et al., 2009). Genes and isoform expression levels were quantified by the RSEM software package. We used the FPKM method to calculate the gene expression levels (Mortazavi et al., 2008). The FDR was computed by dividing the number of falsely discovered genes at a given p-value by the number of statistically significant differentially expressed genes comparing the sample to the control at the same p-value (Benjamini and Yekutieli, 2005). The criteria used for differentially expressed genes (DEG) were FDR ≤ 0.001 and log2 (FPKM in the test sample/FPKM in the reference) ≥ 1. All the data discussed here have been deposited to GEO at NCBI with the accession number of GSE121084.

### Quantitative Reverse Transcription PCR (qRT-PCR) Analysis

To confirm gene expression levels by qRT-PCR, RNA samples that were subjected to the RNA-sequencing were used as templets and the qRT-PCR analysis were performed based on SYBR Green I fluorescence using Roche Light Cycler 480. RNA samples from the uninfected, 201- or 141-infected lymphocytes collected at the 2, 4 and 8 hpi were used as templates with primer pairs to amplify the corresponding target genes (Supplementary Table 2). Correlations between the sequencing data and the qRT PCR results were calculated using linear regression methodology.

### Cytokine Analysis

The cytokines present in the culture supernatants from human lymphocytes infected with 201 or 141 strains for 2, 4, and 8 h were detected using the BD™ Cytometric Bead Array (CBA) Human Inflammatory Cytokine Kit and Human Th1/Th2/Th17 Kit (BD Biosciences, San Jose, CA). The cytokines concentrations were measured by a Becton-Dickinson FACS Caliber flow cytometer. Cytokine levels were processed using Graphpad Prism 5.0, and the statistical significance of the differences in the cytokine levels between the different groups of samples were analyzed by two-way ANOVA analysis followed by Bonferroni post-tests.

### Survival Capabilities of *Y. pestis* Strain 201 and 141 in U937 Cell and RAW264.7 Cells

U937 and RAW264.7 cells were maintained in RPMI 1640 medium containing 10% FBS and 2 mmol/L of L-glutamine at 37°C in a 5% CO_2_ incubator. U937 cells were primed with PMA (100 ng/mL) for 48 hours before infection. U937 and RAW264.7 cells were seeded onto 24-well plates at a concentration of 4 × 10^5^/mL the day before infection. *Y. pestis* strains 201 and 141 were grown in BHI until they reached OD_600nm_ = 1, and each were collected separately by centrifugation and resuspendend in RPMI 1640. The U937 cells or RAW264.7 cells were then infected with strains 201 or 141 at an MOI of 1 or 5, and gentamycin at 100 μg/ml was added to the cells to kill the extracellular bacteria after 0.5 h of infection. At 0.5, 2, 4, 8, 12, 20, 32 hpi, the culture medium from each well was decanted, the cells were thoroughly washed in phosphate-saline buffered saline (PBS), and the infected cells were lysed by addition of sterile H_2_O containing 0.1% Triton X-100 for 15 min at room temperature to release the engulfed bacteria. The living bacteria were counted by plating the diluted cell lysate onto to the agar plates in triplicate. This experiment was performed independently three times as biological triplicates and a representative result is shown as the mean ± SD (*n* = 3).

### Immunoblotting Detection of Proteins Involved in Lysosome Pathway in U937 Cell and RAW264.7 Cells

U937 cells were PMA-primed before they were infected with the *Y. pestis* strain as described above. U937 cells and RAW264.7 cells were maintained, seeded and infected with *Y. pestis* strains 201 and 141 at an MOI = 10, according to the method described in the survival capability assays. Gentamycin was added to the cultures to inhibit the over proliferation of the bacteria at 0.5 hpi. U937 and RAW264.7 cells were harvested at 2, 4, and 8 hpi, washed with ice-cold PBS, and lysed with lysis buffer (50 mM Tris-HCl [pH 7.4], 150 mM NaCl, 1% TritonX-100, 1% sodium deoxycholate, 2 mM sodium pyrophosphate, 1 mM EDTA) supplemented with a protease inhibitor complete cocktail (Roche, Basel, Switzerland). Sodium dodecyl sulfate (SDS) loading buffer was added into the cell lysate, which was boiled and then separated by 12% SDS-polyacrylamide gel electrophoresis (SDS-PAGE), followed by transfer onto a polyvinylidene fluoride membrane (GE Healthcare, Piscataway, NJ, USA). Specific proteins were detected using antibodies against Actin, CTSZ, CTSD and SLC11A1(Santa Cruz Biotechnology, Dallas, TX).

## Discussion

*Yersinia pestis* has evolved from *Y. pseudotuberculosis*, a foodborne enteropathogenic pathogen responsible for self-limiting intestinal infections (Morelli et al., 2010). Three pathogenic *Yersinia* bacteria shared a common 70-Kb virulence plasmid that encodes the type three secretion system (T3SS), which can deliver several *Yersinia* virulence effectors called *Yersinia* outer membrane proteins (Yops) into the eukaryotic cell cytosol to paralyze host defenses (Cornelis, 2002). Acquiring pPCP1 and pMT1 plasmids has endowed *Y. pestis* with new virulent factors such as the F1 capsular antigen, the Pla plasminogen activator, and the murine toxin, all of which are critical for this bacterium to be a flea-transmitted lethal pathogen (Prentice and Rahalison, 2007). Massive gene losses have also played very important roles in the evolution of *Y. pestis* (Chain et al., 2004). Indeed, several newly acquired features contributing to the high pathogenicity and flea-bite transmissibility of *Y. pestis* have resulted from reductive evolution. For instance, the Inv and YadA adhesins which are needed for enteropathogenic yersiniae colonization the Peyer's patches and mesenteric lymph nodes, are both pseudogenes in *Y. pestis* (Marra and Isberg, 1997; Parkhill et al., 2001; Heise and Dersch, 2006). Furthermore, with several biosynthesis gene for LPS being inactivated in *Y. pestis*, LPS in this bacterium lacks the O antigen and switches from a hexa-acylated to tetra-acylated form that is poorly recognized by TLR4 (Kawahara et al., 2002; Rebeil et al., 2004). *Y. pestis* Microtus strains are thought to lie on the evolutionarily oldest branch of the *Y. pestis*, which are capable of transmission via fleabites and are also highly virulent in mice but are unable to infect larger mammals such as rabbits, guinea pigs and humans (Fan et al., 1995; Song et al., 2004). Comparative genomic analysis of *Y. pestis* from distinct biovars has found little evidence in their different genome compositions to accounts for the host-specificity displayed by Microtus. Here, we sought to probe into this mystery in the context of the host response and further investigate the interactions between the Microtus strain and human or murine macrophages. Our results indicate that human peripheral lymphocytes respond differently to infections with the *Y. pestis* Microtus strain and the strain virulent for humans after their initial encounter, especially in several innate immunity pathways including the lysosome and TLR signaling pathways (Figure 2). We assumed that the PAMPs that are present on the bacterial surface or the degraded small molecules arising from 201 and 141 bacteria are differentially recognized by human peripheral lymphocytes, leading to the distinct innate immune responses in these cells. Our additional *in vitro* study confirmed that the human macrophages were competent at clearing the biovar Microtus strains of *Y. pestis*, unlike the mouse macrophages where this bacterium was able to bacteria to survive for a much longer time (Figure 5). This result is consistent with the transcriptomic results that gene expressions in the lysosome pathway were significantly enriched in the 201-infected lymphocytes and two functionally important proteins, CTSD and SLC11A1 that are involved in lysosome activation, showed enhanced expression. It has long been established that the initial interaction between the host macrophages and *Y. pestis* bacilli is critical to systemic infection progression, and our findings hint that human and murine macrophages exhibit distinct recognition or clearing abilities when infected with the Microtus strains. This finding is probably related to some uncharacterized features that differs between Microtus and the other biovars strains, or slight but very critical differences in some receptors between the human and mouse hosts. Further investigations are clearly needed in this area, especially for the discovery of the molecular mechanisms underlying the host-specific pathogenesis properties of the *Y. pestis* biovar Microtus strains.

## Author Contributions

ZD, RY, and XZ designed and cooperated the study. QWZ, YX, HZ, and ZQ isolated peripheral lymphocytes and performed the cell infection experiments. RL, QZ, and QWZ performed the survival rates analysis of the *Y. pestis* strains and immunoblotting analysis. XX performed qRT-PCR analysis. ZD, YFY, and ZK analyzed the data. YS, YC, TW, and YY provided technical assistance. ZD and QWZ wrote the manuscript.

### Conflict of Interest Statement

The authors declare that the research was conducted in the absence of any commercial or financial relationships that could be construed as a potential conflict of interest.

## Supplementary Material

Supplementary Figure 1 Correlations between the expression levels of 23 genes measured by RNA-seq and qRT-PCR were analyzed using the linear regression method.

Supplementary Figure 2 Several important molecules in the TLR signaling pathway were differentially expressed in the 201- and 141-infected human lymphocytes. Changes in the expression levels of CD14, IRF5, TLR8, and TICAM2 in 201- and 141-infected lymphocytes were plotted against the time point from infection initiation according to the RNA-seq analysis results **(A)**. Expression changes for the same group of genes were plotted according to the qRT-PCR results **(B)**.

Supplementary Figure 3 Several important molecules in the lysosome pathway were differentially expressed in the 201- and 141-infected human lymphocytes. Changes in the expression levels of ATP6V0A1, CTSD, CTSK and SLC11A1 in the 201- and 141-infected lymphocytes were plotted against the time point from infection initation according to the RNA-seq analysis results **(A)**. Expression changes for the same group of genes were plotted according to the qRT-PCR results **(B)**.

Supplementary Table 1 Primers used for qRT-PCR.

Supplementary Table 2 Correlation coefficients between RNA-seq analysis and q-RT PCR results were analyzed using the samples from the lymphocytes infected with strain 201 and the normal cell control.
