# Supplementary figures and images for: Human Macrophages Clear the Biovar Microtus Strain of Yersinia pestis More Efficiently Than Murine Macrophages
Source: Front Cell Infect Microbiol. 2019 Apr 24;9:111. doi: 10.3389/fcimb.2019.00111 (PMC6491462; doi:10.3389/fcimb.2019.00111)

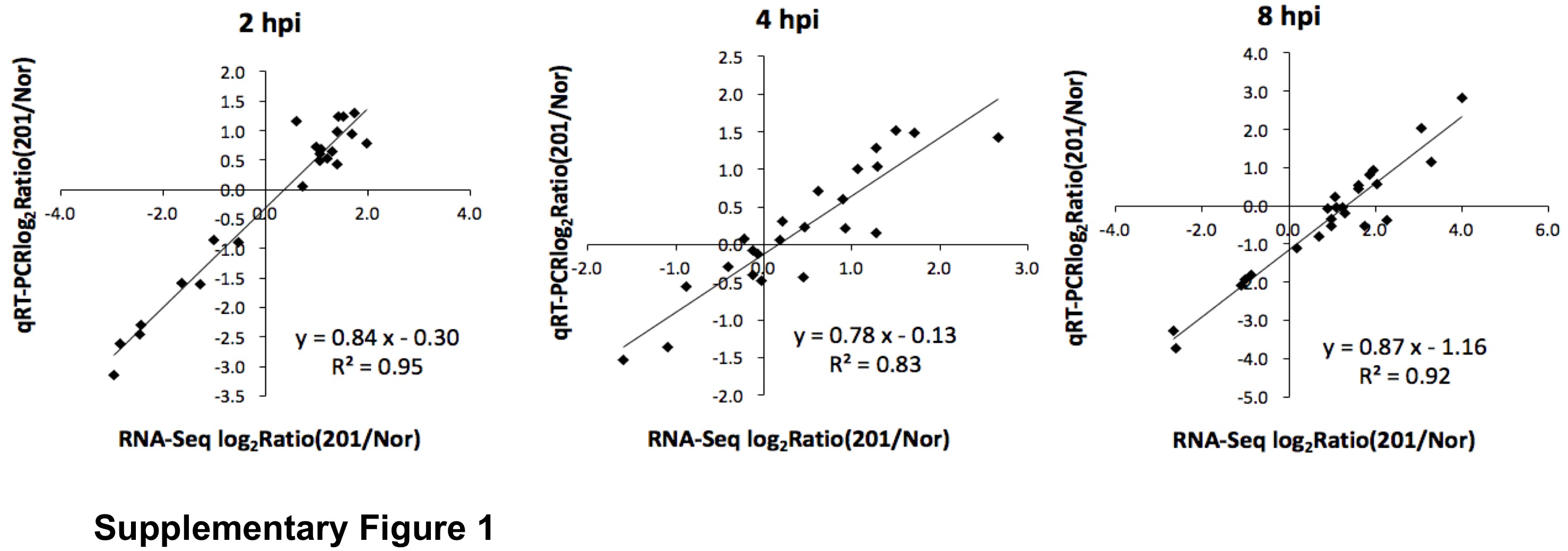

Supplement: Supplementary Figure 1 — Correlations between the expression levels of 23 genes measured by RNA-seq and qRT-PCR were analyzed using the linear regression method. [file Image_1.JPEG]

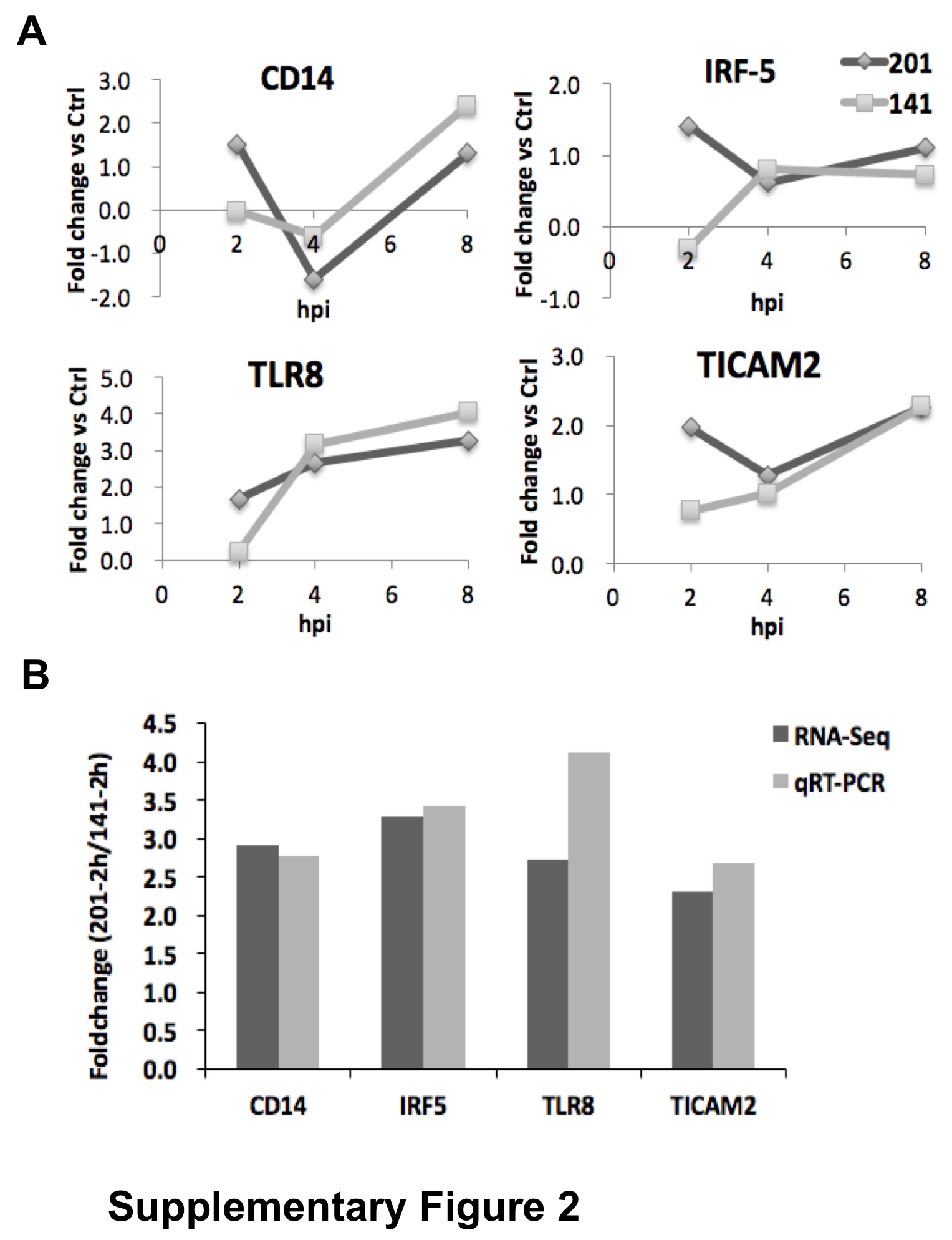

Supplement: Supplementary Figure 2 — Several important molecules in the TLR signaling pathway were differentially expressed in the 201- and 141-infected human lymphocytes. Changes in the expression levels of CD14, IRF5, TLR8, and TICAM2 in 201- and 141-infected lymphocytes were plotted against the time point from infection initiation according to the RNA-seq analysis results (A). Expression changes for the same group of genes were plotted according to the qRT-PCR results (B). [file Image_2.JPEG]

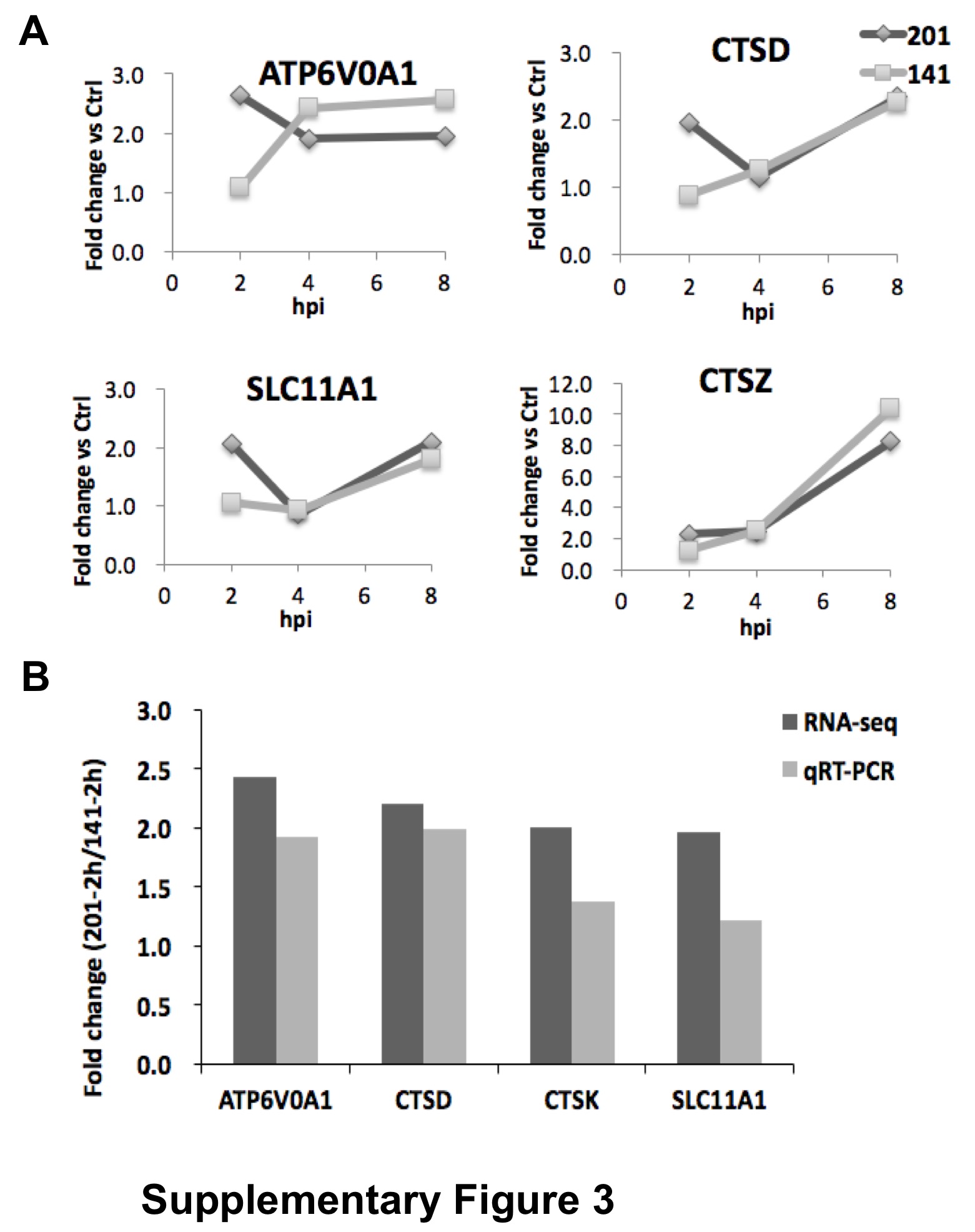

Supplement: Supplementary Figure 3 — Several important molecules in the lysosome pathway were differentially expressed in the 201- and 141-infected human lymphocytes. Changes in the expression levels of ATP6V0A1, CTSD, CTSK and SLC11A1 in the 201- and 141-infected lymphocytes were plotted against the time point from infection initation according to the RNA-seq analysis results (A). Expression changes for the same group of genes were plotted according to the qRT-PCR results (B). [file Image_3.JPEG]
